# Protein engineering of transaminase facilitating enzyme cascade reaction for the biosynthesis of azasugars

**DOI:** 10.1016/j.isci.2024.109034

**Published:** 2024-01-26

**Authors:** Yueming Zhu, Peng Chen, Qianzhen Dong, Qian Li, Dechuan Liu, Tao Liu, Weidong Liu, Yuanxia Sun

**Affiliations:** 1 Tianjin Institute of Industrial Biotechnology, Chinese Academy of Sciences, National Technology Innovation Center of Synthetic Biology, Tianjin 300308, China; 2 University of Chinese Academy of Sciences, Beijing 100049, China

**Keywords:** Chemistry, Chemical engineering, Organic chemistry, Organic synthesis

## Abstract

Azasugars, such as 1-deoxynojirimycin (1-DNJ), exhibit unique physiological functions and hold promising applications in medicine and health fields. However, the biosynthesis of 1-DNJ is hindered by the low activity and thermostability of the transaminase. In this study, the transaminase from *Mycobacterium vanbaalenii* (MvTA) with activity toward d-fructose was engineered through semi-rational design and high-throughput screening method. The final mutant M9-1 demonstrated a remarkable 31.2-fold increase in specific activity and an impressive 200-fold improvement in thermostability compared to the wild-type enzyme. Molecular dynamics (MD) simulations revealed that the mutation sites of H69R and K145R in M9-1 played crucial roles in the binding of the amino acceptor and donor, leading to the stable conformation of substrates within the active pocket. An enzyme cascade reaction was developed using M9-1 and the dehydrogenase from *Paenibacillus polymyxa* (GutB1) for the production of mannojirimycin (MJ), which provided a new idea for the *in vitro* biosynthesis of 1-DNJ.

## Highlights

• Enzyme engineering of MvTA highly improved its activity and thermostability
• High-resolution crystal structures of MvTA mutant were obtained and resolved
• The crucial role of mutation sites H69R and K145R in active pocket was revealed
• Enzyme cascade reaction was developed for the *in vitro* production of 1-DNJ precursor

## Graphical abstract

**Figure:**
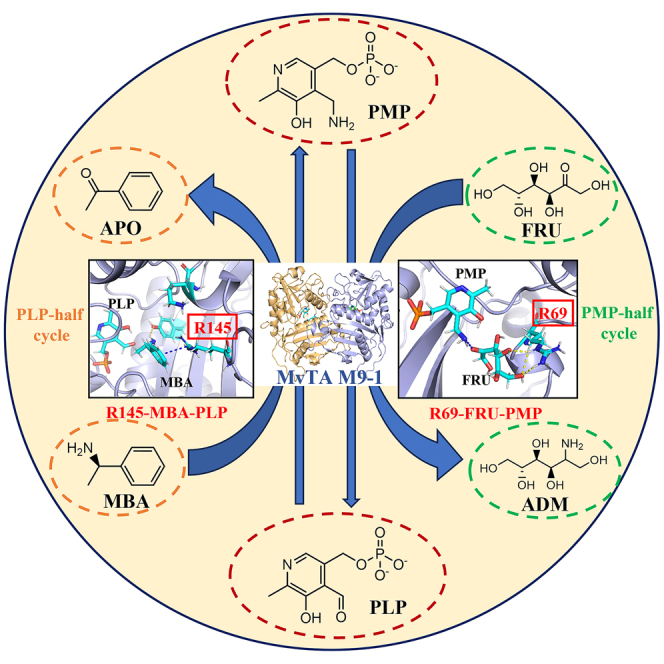

## Introduction

Azasugars are sugar analogues in which one or more oxygen atoms in the sugar ring are replaced by nitrogen. Due to their structural similarity to common sugars, these compounds and their derivatives exhibit potent inhibitory activity against glycosidases, thus impeding carbohydrates decomposition and influencing the modification of oligosaccharide chains in glycolipid and glycoprotein biosynthesis.^1^ Consequently, azasugars hold promise as therapeutic agents for glucose metabolism-related disorders.^2^^,^^3^^,^^4^

One particular azasugar of significant interest is 1-deoxynojirimycin (1-DNJ). As a glucose analogue, it can competitively bind to glucosidase, inhibiting its activity and preventing the breakdown of polysaccharides into monosaccharides.^5^ Therefore, 1-DNJ and its derivatives demonstrate considerable potential for the prevention and treatment of diabetes.^6^ Miglitol, a 1-DNJ derivative, is a commonly used anti-diabetic drug.^7^ Moreover, 1-DNJ and its derivatives have demonstrated anti-cancer, anti-inflammatory and anti-viral effects.^8^^,^^9^^,^^10^

Chemical synthesis methods for 1-DNJ tend to be cumbersome, inefficient, and environmentally unfriendly. However, certain organisms have the inherent capacity to synthesize 1-DNJ. For instance, the abundant presence of 1-DNJ in mulberry leaves suggested the existence of a biosynthetic pathway for 1-DNJ in mulberry.^11^^,^^12^ Additionally, microorganisms such as *Bacillus subtilis*, *Bacillus amyloliquefaciens* and *Paenibacillus polymyxa* can produce 1-DNJ.^13^^,^^14^^,^^15^ Clark et al. identified key genes involved in the synthesis of 1-DNJ in *B*. *amyloliquefaciens*, confirming that the *gabT1*, *yktC1* and *gutB1* genes encode transaminase, phosphatase, and dehydrogenase, respectively.^16^ In the microbial synthetic pathway of 1-DNJ, fructose 6-phosphate acts as the amino receptor, and its transamination product undergoes a series of catalytic reactions to yield 1-DNJ as the final product. Evidently, the transaminase is essential for 1-DNJ biosynthesis.

The transaminase utilized for 1-DNJ biosynthesis is an ω-transaminase widely employed as a biocatalyst for chiral amines synthesis.^17^ Nevertheless, the wild-type transaminase exhibits limitations in terms of substrate specificity, stability, and catalytic efficiency, necessitating enzyme engineering to meet industrial requirements. For instance, the specific activity of GabT1 from *Paenibacillus polymyxa* SC2 is merely 4.9 nmol/min/mg, resulting in extremely low efficiency in producing 1-DNJ with this strain.^15^ Rational design and directed evolution strategies have been employed to improve properties of various transaminases, and high-throughput screening methods for transaminases have been developed.^18^^,^^19^^,^^20^

In this study, we focus on the ω-transaminase from *Mycobacterium vanbaalenii* (MvTA) which exhibits activity toward d-fructose rather than fructose 6-phosphate. Unlike GabT1, MvTA offers the advantage of producing transamination product that do not necessitate dephosphorylation by a phosphatase, thereby streamlining the synthesis pathway of 1-DNJ. Considering the low enzymatic activity of MvTA, a rapid and sensitive high-throughput screening strategy based on colorimetric assay was developed for enzyme engineering. A mutant M9 with significantly enhanced activity and thermostability has been successfully obtained and crystallized for structural resolution. Further improvement in specific activity was achieved through the utilization of high-resolution structure and computer-aided rational design approach. Molecular docking and molecular dynamics (MD) simulations have been conducted to elucidate the mechanism underlying the improved activity. Considering the enzyme’s ability to directly transfer amino group to d-fructose, an *in vitro* enzyme cascade reaction has been established for the production of azasugars. The best MvTA mutant, in conjunction with the dehydrogenase from *Paenibacillus polymyxa* (GutB1), has been employed as biocatalysts to produce mannojirimycin (MJ), the precursor of 1-DNJ, using d-fructose as the substrate. Leveraging the efficient catalytic activity of MvTA mutant on various hexaketoses, a series of azasugars can be synthesized and utilized in the preparation of end products with desirable biological functions.

## Results and discussion

### Development of high-throughput screening method

**Figure 1:**
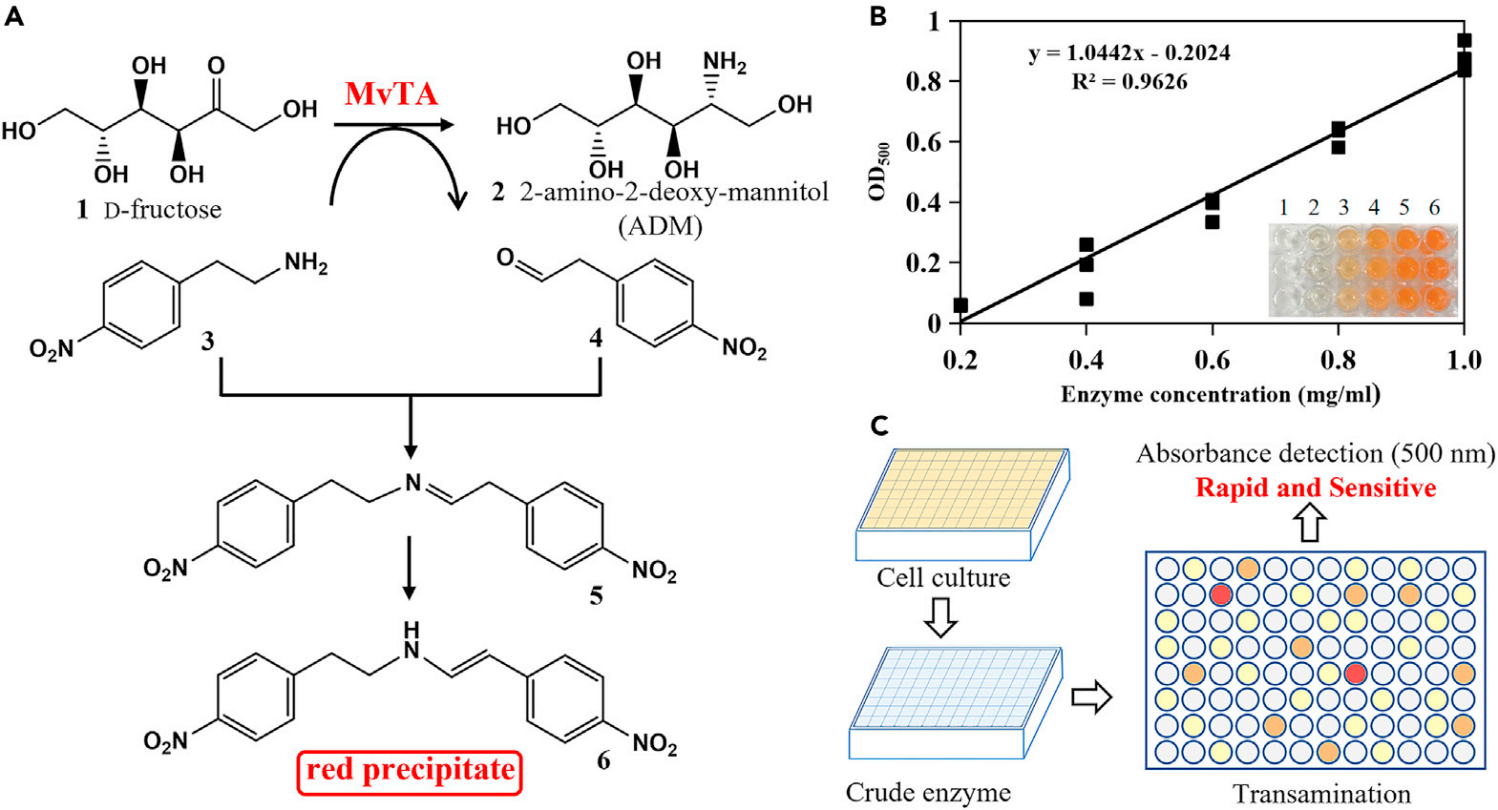
Establishment of a high-throughput screening method (A) Colorimetric measurement principle of transaminase activity using d-fructose as the amino acceptor. (B) Standard curve correlating absorbance values at 500 nm with enzyme activity. (C) Workflow of the high-throughput screening process.

The transaminase MvTA demonstrates transaminate activity toward d-fructose **1**, and the resulting 2-amino-2-deoxy-mannitol (ADM) **2** undergoes dehydrogenation under the enzyme GutB1 and spontaneously cyclizes to produce the valuable azasugar mannojirimycin (MJ), which serves as a precursor for 1-DNJ. However, the application performance of MvTA is hindered by its low activity and poor thermostability. To address these limitations, enzyme engineering is required to enhance its performance. Previous reports indicated that MvTA could catalyze the transamination reaction using d-fructose as the amino acceptor and 2-(4-nitrophenyl)ethan-1-amine **3** as the amino donor.^21^ In the reaction, **3** was converted into the corresponding aldehyde **4**, which subsequently formed an imine **5** with **3**. After tautomerization, a red precipitate **6** is produced (Figure 1A). The transamination reaction was conducted at different concentrations of MvTA, and the absorbance of the product was measured at 500 nm. A clear linear relationship was observed between the absorbance of the red precipitate product (OD_500_) and the amount of enzyme added (Figure 1B), indicating that OD_500_ can serve as a measure of enzyme activity. Based on the principle, a high-throughput screening method for mutants was established. Single clones from mutant libraries were cultivated in 96-deep-well plates. Cells were disrupted using freeze-thawing method to obtain supernatants, which were transferred to the 96-well plates for the transamination reaction. The product was tested using a colorimetric assay, and mutants exhibited higher OD_500_ values (indicating a dark red color) were rapidly and sensitively screened (Figure 1C).

### Enzyme engineering to improve activity and thermostability

The properties of enzyme MvTA were characterized, revealing that its optimal temperature and pH were 50°C and 6.0, respectively. The enzyme activity was independent of metal ions. However, MvTA displayed low activity toward d-fructose, with an activity level of 2.4 ± 0.3 mU/mg under optimal conditions. Furthermore, MvTA exhibited extremely poor thermostability, with more than 80% activity loss after incubation for 1 h at 50°C (Figure S1). To gain a better understanding of the enzyme and facilitate its engineering, the three-dimensional structure of MvTA was obtained through homology modeling using Swiss-Model server. The transaminase from *Arthrobacter* sp. KNK168 (3WWI) was used as the template,^22^ which showed 50.66% similarity with MvTA. The predicted structure of MvTA revealed a homodimeric arrangement with PLP (pyridoxal phosphate) as the coenzyme. The active pocket was situated at the interface of two chains of the homodimer.

**Figure 2:**
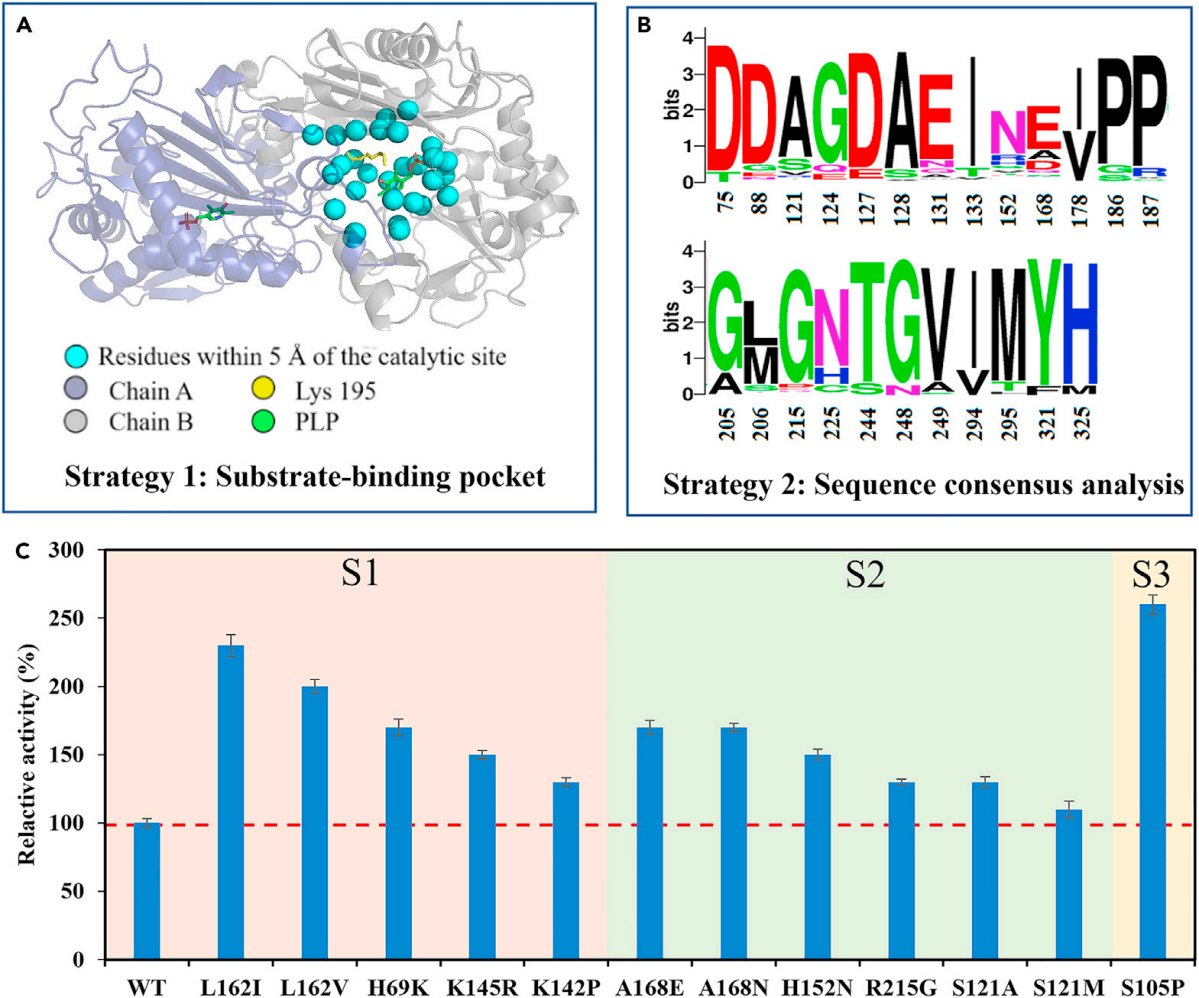
Strategies for constructing site-saturation mutagenesis libraries and generating single-site mutants with enhanced activity (A) Strategy 1: saturation mutagenesis targeting amino acid sites within 5 Å of the catalytic site (K195) and PLP. (B) Strategy 2: saturation mutagenesis of conserved amino acid sites identified through consensus analysis. (C) Single-site mutants with improved activity. S1, S2, and S3 represented for Strategy 1, Strategy 2, and Strategy 3, respectively.

Three strategies were employed for MvTA engineering. Firstly, based on the predicted structure of MvTA, 34 residues within a 5 Å range of catalytic site (Lys195) and PLP were selected for site saturation mutagenesis (Figure 2A). The second strategy involved the selection of mutation sites using the consensus analysis approach.^23^^,^^24^ A total of 250 transaminases exhibiting high similarity to MvTA were retrieved from the Uniprot database for multiple sequence alignment. Based on the alignment results, 24 candidate residues were identified for site saturation mutagenesis (Figure 2B). In total, site saturation mutagenesis libraries were constructed for 58 residues. Additionally, a random mutagenesis library was constructed using random PCR approach. The activities of individual clones from mutagenesis libraries were assessed using the high-throughput screening method, and the mutants exhibited increased activity were further confirmed using HPLC.

As a result, a total of 12 site-directed mutants with increased activity were identified from more than 6,000 individual clones (Figure 2C). It was observed that the residues located within the substrate binding pocket, such as L162, H69, and K145, significantly influenced the activity of MvTA. The mutant K142P suggested that the residues within the substrate channel might be involved in enzyme activity. Additionally, six mutants (A168E, A168N, H152N, R215G, S121A, and S121M) obtained through Strategy 2 showed increased activity compared to the wild type (WT). Furthermore, the random mutagenesis approach yielded the S105P mutant, which displayed a significant increase in activity.

The relative thermostability of the single mutants were evaluated. The results suggested that the mutations in residues surrounding the substrate binding pocket (L162I, H69K, K145R and K142P) had minimal impact on thermostability, while the mutations in residues positioned on the surface (S105P, S121M, H152N, A168E, and R215G) notably enhanced thermostability (Figure S2). The residues H152, A168 and R215 were mutated to the highly conserved amino acids based on sequence consensus analysis, indicating the significant contribution of natural evolution to the thermostability of the enzyme.^25^

**Figure 3:**
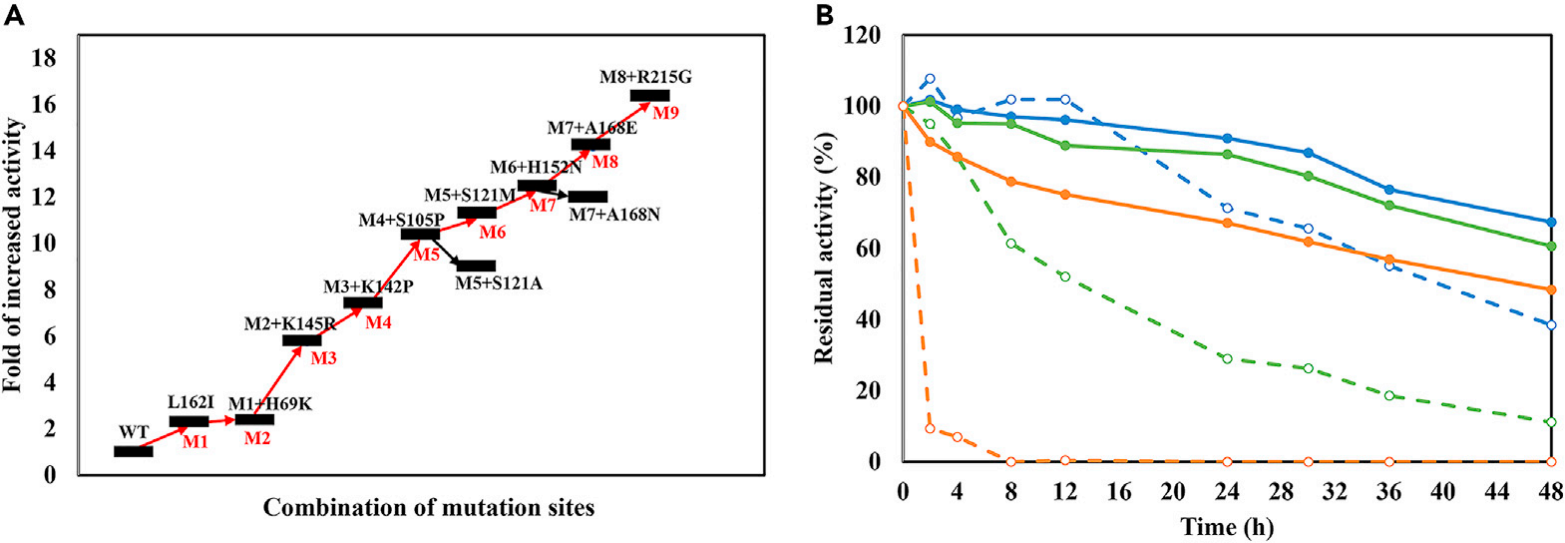
Ehancement of specific activity and thermostability in combinatorial mutants (A) Specific activities of the combinatorial mutants. (B) Residual activity of M9 (solid line) and WT (dashed line) after incubated at 30 (blue), 40 (green), and 50°C (orange).

The mutation sites within the substrate binding pocket and the substrate channel were combined to generate M2, M3, and M4, which exhibited progressively increased activity. Based on the level of thermostability improvement, the additional five mutation sites (S105P, S121M, H152N, A168E and R215G) were sequentially incorporated into M4, resulting in the generation of mutant M9 (Figure 3A; Table 1). The activity of M9 was measured as 39.4 ± 1.5 mU/mg, demonstrating a remarkable 16.4-fold increase compared to that of WT. Notably, M9 exhibited significant enhancement in thermostability compared to WT (Figure 3B). The half-life (t_1/2_) of M9 at 50°C represented a 208-fold improvement compared to WT (Table 2). Additionally, the results indicated that the optimal pH of M9 remained unchanged compared to WT, while the optimum temperature was elevated from 50°C to 55°C (Figure S3).

**Table 1:** Specific activities of MvTA mutants relative to WT

Enzymes	Mutation sites	Activities (mU/mg)	fold
WT	–	2.4	1.0
M1	L162I	5.5	2.3
M2	L162I/H69K	5.8	2.4
M3	L162I/H69K/K145R	13.8	5.8
M4	L162I/H69K/K145R/K142P	17.8	7.4
M5	L162I/H69K/K145R/K142P/S105P	25.0	10.4
M6	L162I/H69K/K145R/K142P/S105P/S121M	27.1	11.3
M7	L162I/H69K/K145R/K142P/S105P/S121M/H152N	30.0	12.5
M8	L162I/H69K/K145R/K142P/S105P/S121M/H152N/A168E	34.1	14.2
M9	L162I/H69K/K145R/K142P/S105P/S121M/H152N/A168E/R215G	39.4	16.4

**Table 2:** Half-lives of purified WT and mutant M9 at different temperature

Temperature (°C)	t_1/2_ of WT (h)	t_1/2_ of M9 (h)	Fold
30	41.84	175.13	4.19
40	13.44	119.07	8.86
50	0.22	45.82	208
60	NA	3.04	NA

### Crystal structure of MvTA wildtype and mutant

The crystal structures of the WT (PDB: 8IOZ), M9-PLP (PDB: 8ISC) and M9-PLP-Fru (PDB: 8IVP) were resolved at resolutions of 2.33 Å, 2.27 Å, and 1.93 Å, respectively (Table S2). However, four critical loops (Loop1: residues 138–149, Chain A; Loop2: residues 187–197, Chain A; Loop3: residues 136–150, Chain B; Loop4: residues 186–197, Chain B) were missing in 8IOZ (Figure S4). These loops contained essential residues for enzyme activity. For insatance, the Lys195, located on Loop2 (Chain A) and Loop4 (Chain B), served as the catalytic residue, and the flexible loops of Loop1 and Loop3 were likely involved in substrate binding. Li et al. reported the removal of the loop backbone (Arg134-Leu146) from the substrate tunnel entrance in the transaminase from *Rhodobacter* sp. 140A (RbTA) resulting in an “open” conformation.^26^ In contrast, the loops of (R)-transaminases, such as MvTA, mostly lined the substrate tunnel entrance in a “closed” conformation.

**Figure 4:**
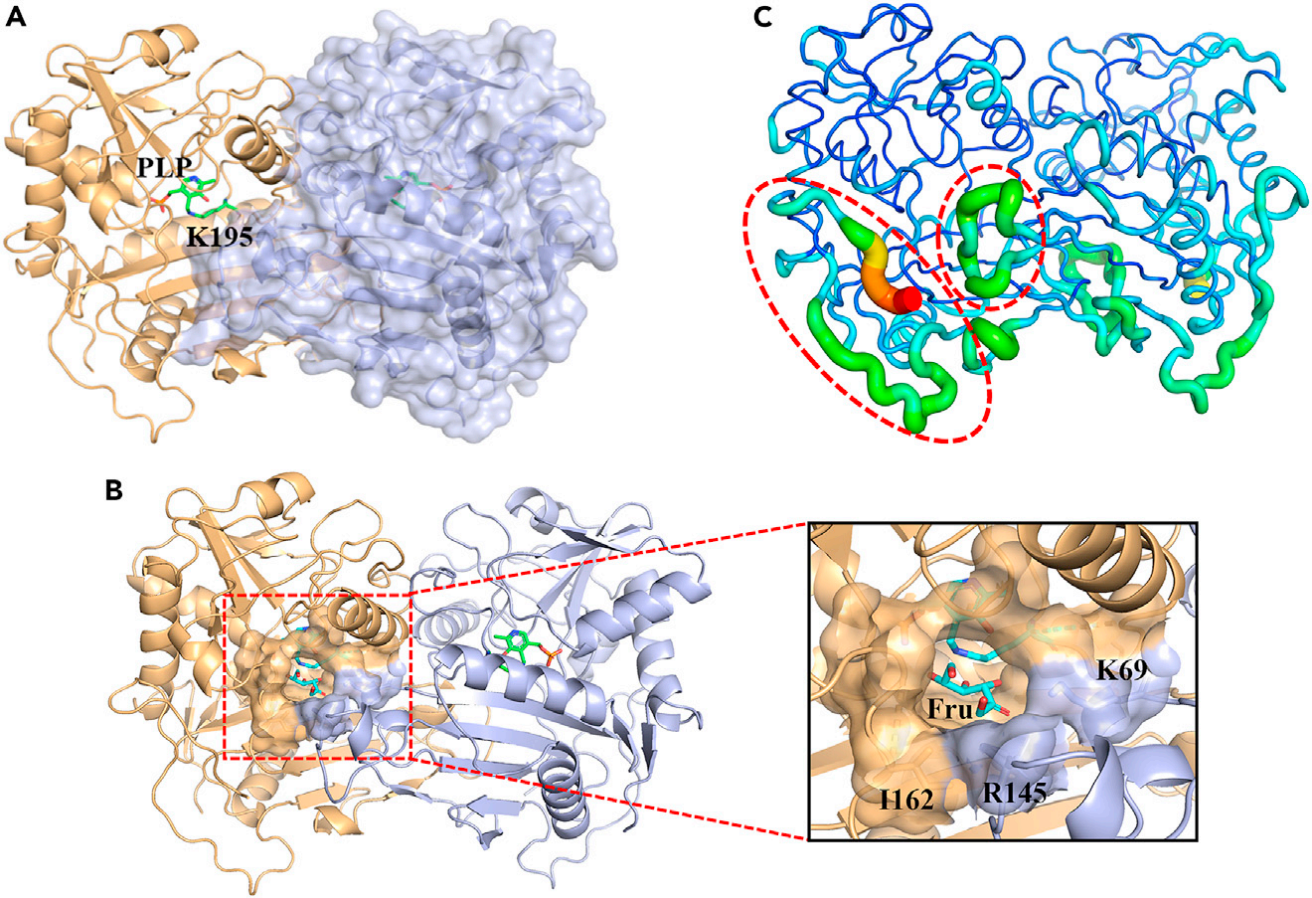
Structure analysis of the M9 mutant (A) Overall structure of M9-PLP (PDB ID: 8ISC) complex highlighting the covalent bond between K195 and PLP. (B) Overall structure of M9-PLP-Fru (PDB ID: 8IVP) complex illustrating the binding both PLP and d-fructose, indicating the presence of a binding pocket at the interface of two subunits. (C) B-factor analysis of the M9 structure, providing insights into the flexibility of the protein.

The structure of 8ISC and 8IVP displayed complete overall conformations except for the N-loop, suggesting that the mutation sites in M9 contributed to stabilizing the overall conformation of the enzyme and facilitating the acquirement of the complex structure. In all three structures, the asymmetric unit comprised two polypeptide chains forming a homodimer. The dimeric enzyme exhibited the characteristic structural architecture of class IV PLP-dependent enzymes, consisting of two subunits (Figure 4A). The interface between the two subunits constituted the binding pocket for the amino donor and acceptor (Figure 4B). Coenzyme PLP formed a Schiff base linkage with the catalytic residue Lys195 in the complex structures of 8ISC and 8IVP, consistent with previously research.^27^ B-factor analysis of 8ISC revealed pronounced flexibility in the loop region (Y138−T151) (Figure 4C). Upon comparing the B-factor values between 8ISC and 8IVP using the B-FITTER software,^28^ it was observed that the overall B-factor values of 8IVP were lower than those of 8ISC (Figure S5). Particularly, Arg145 in 8IVP exhibited a significantly lower B-factor value than in 8ISC. The results suggested that fructose binding played a role in stabilizing the protein conformation and hinted at a potential interaction between fructose and the residue Arg145.

### Molecular basis of enhanced activity

Molecular docking was performed to generate the crystal structure of M9-PLP-MBA and WT-PLP-MBA. Molecular dynamics (MD) simulations of these two complex structures were carried out using GROMACS. During the 100 ns trajectory, the distances between the N atom of (R)-MBA and O atom of aldehyde group on PLP (d_MBA-PLP_) were measured (Figure S6A). It was observed that the value of d_MBA-PLP_ reached a stable level within the last 80 ns trajectory of M9 complex trajectory, whereas it exhibited fluctuated throughout the 100 ns trajectory of WT complex. Additionally, the average value of d_MBA-PLP_ for M9 was significantly smaller than that for WT. Trajectory analysis suggested that (R)-MBA displayed substantial swinging motion within the substrate binding pocket of WT. Hydrogen bond analysis revealed frequent formation of hydrogen bonds between (R)-MBA and H69 during the 100 ns trajectory of WT (Figure S6B), whereas hydrogen bonds between (R)-MBA and K69 in M9 were scarcely observed (Figure S6C).

**Figure 5:**
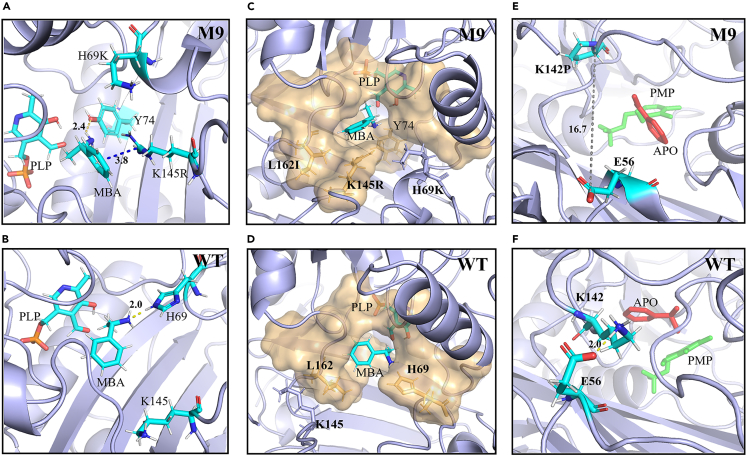
The molecular basis of enhanced enzyme activity by mutation sites Structure comparison between M9 (A, C and E) and WT (B, D and F) illustrating the molecular basis of enhanced activity. (A) M9: π-cation interaction between R145 and the benzene ring of (R)-MBA, absence of hydrogen bond between K69 and (R)-MBA. (B) WT: hydrogen bond between H69 and (R)-MBA, resulting in greater distance between the amino donor and PLP. (C) M9: tight binding of (R)-MBA within the active pocket due to the synergistic effect of I162 and R145 in the complex structure. (D) WT: (R)-MBA potentially swings within the active pocket due to a larger active pocket. (E) M9: absence of hydrogen bonds between the sidechain of P142 and E56, facilitating the exit of APO and entry of d-fructose. (F) WT: the hydrogen bond interaction between K142 and E56 may close the channel for substrate entry and exit.

Representative structures from 90 to 100 ns trajectory were selected for further analysis, revealing the occurrence of a π-cation interaction between the N atom of the guanidine group of R145 and the benzene ring of (R)-MBA in M9 complex (Figure 5A). Furthermore, due to the mutation of H69K, the longer sidechain of lysine rotated away from (R)-MBA due to electrostatic repulsion by R145, thereby preventing the formation of a hydrogen bond with (R)-MBA (Figures 5A and 5B). Further analysis of the binding pocket demonstrated that the mutation of L162I stablized the benzene ring on (R)-MBA within the pocket (Figures 5C and 5D). Through the combined effects of the mutation sites K69, R145 and I162, (R)-MBA approached PLP in a stable conformation, thereby promoting the efficiency of the first half-reaction of transamination.

To elucidate the second half-reaction of transamination, molecular docking of the ligand pyridoxamine phosphate (PMP) and acetophenone (APO) were performed in both WT and M9 to generate the complex structures WT-PMP-APO and M9-PMP-APO, respectively, for MD simulation. Trajectory analysis and examination of representative structures from 90 to 100 ns trajectory revealed that hydrogen bonds were frequently formed between K142 and E56 in WT. However, due to the mutation of K142P in M9, the sidechain of proline was unable to form any hydrogen bonds with E56 (Figures 5E and 5F). As a result, a channel was opened for the release of APO and the entry of d-fructose, potentially further enhancing the catalytic efficiency of MvTA. Likewise, in the M9-PLP-MBA system, there was no formation of hydrogen bond between P142 and E56, as indicated by an average distance of 8.39 Å between these residues.

Moreover, the mutation of K142P notably heightened the rigidity of the loop region (137–146). MD simulations of the M9-PLP-MBA complex highlighted lower RMSF values for this loop region in M9 compared to WT (Figure S7). The increased rigidity in the loop region significantly contributed to the stabilization of R145, consequently enhancing the stability of (R)-MBA within the substrate pocket. Many studies reported the role of proline in enhancing stability within loop regions.^29^^,^^30^ The S105P mutation exhibited a similar situation. In both the first and second half-reactions, the RMSF values for the loop region (102–108) and its adjacent α-helices in M9 were notably lower compared to WT (Figure S7). These findings indicated that the proline substitution at position 105 could enhance the rigidity of the loop region, consequently reducing the flexibility of its neighboring α-helices. This increased rigidity significantly contributed to the thermostability of the enzyme, thereby resulting in enhanced activity.

### Further enhancement of activity through rational design

**Figure 6:**
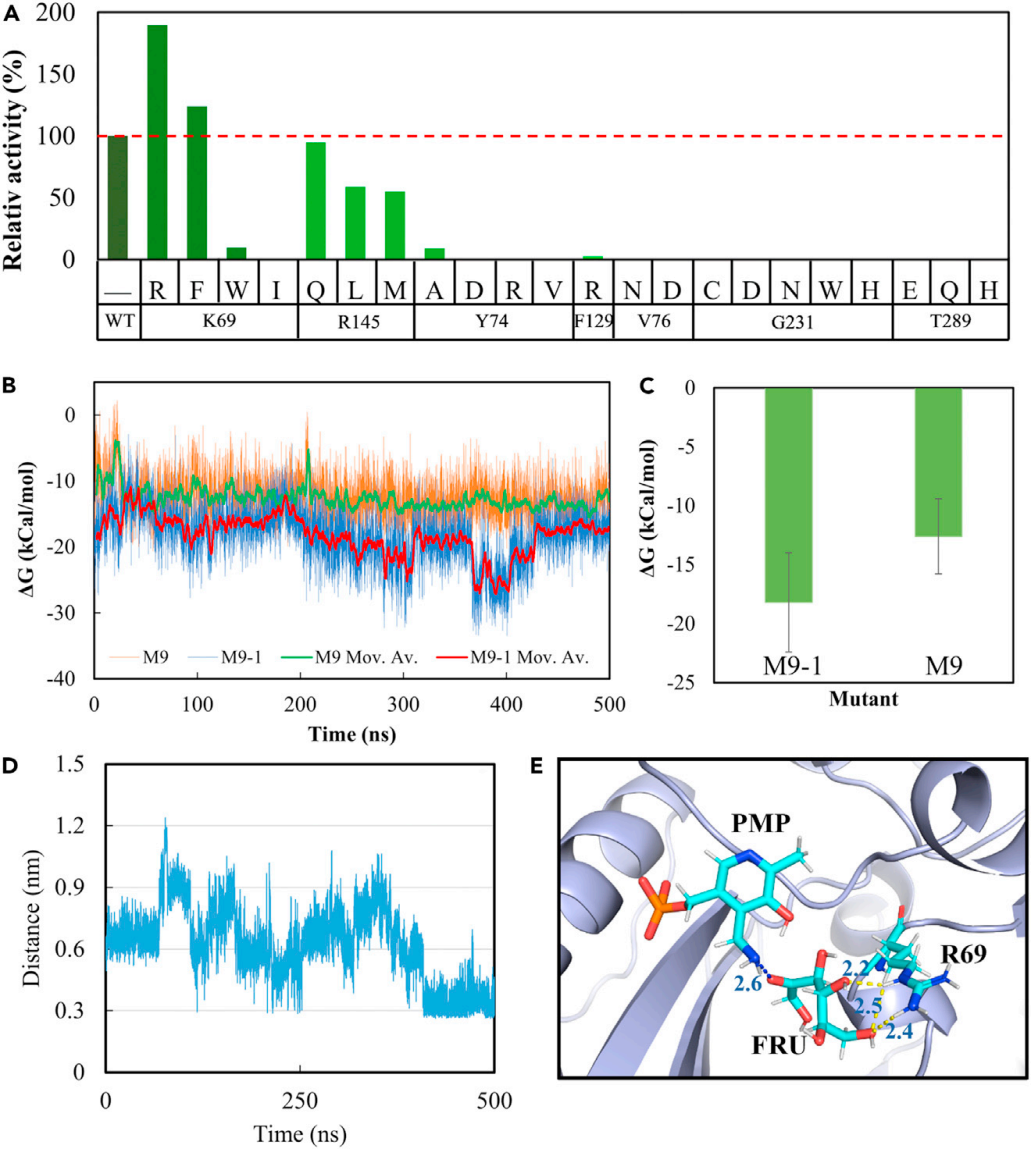
Further enhancement in MvTA activity through rational design (A) Relative activities of mutations exhibiting ΔΔG < −1.0, caculated using the Cartesian_ddg application. (B) Moving average values (Mov. Av.) of binding free energy between d-fructose and M9 or M9-1. (C) Average values of binding free energy between d-fructose and M9 or M9-1. (D) Distances between the N atom on amino group of PMP and the O atom on ketone group of d-fructose during the 500 ns trajectory. (E) Hydrogen bond between R69 and d-fructose in M9-1.

To further enhance the activity of M9, a computational design approach was employed based on the high-resolution crystal structure (8IVP). Six specific residues (K69, Y74, V76, F129, R145, and T289) were selected as targets for mutation using the Cartesian_ddg application. The results revealed that residues Y74, V76, F129, and T289 played crucial roles in enzyme activity, as mutations at these positions completely abolished the activity. In contrast, residues K69 and R145 exhibited some variability, with their mutations leading to both increased and decreased activities. Notably, the mutant K69R (M9-1) demonstrated an impressive nearly 2-fold improvement in activity compared to M9 (Figure 6A). The half-lives (t_1/2_) of M9-1 at 30, 40, and 50°C were 172.8, 122.5, and 44.3 h, respectively, showing no significant change compared to those of M9.

The kinetic parameters of WT, M9, and M9-1 toward (R)-MBA and d-fructose were determined and presented in Table 3. The kinetic parameters of WT toward d-fructose could not be reliably determined because of its extremely low activity. Notably, the *K*_*m*_ value toward d-fructose of M9-1 was found to be smaller than that of M9, suggesting a higher affinity of protein for the amino acceptor. The significantly increased number of hydrogen bonds between d-fructose and M9-1 compared to WT also indicated the enhancement in fructose affinity. (Figure S8). Furthermore, The gmx_MMPBSA approach was employed to calculate the binding free energy between the amino acceptor and the protein. The results revealed that both the moving average (Mov. Av.) and average values of binding free energy between d-fructose and M9-1 were consistently lower compared to those between d-fructose and M9 throughout 500 ns trajectory (Figures 6B and 6C). The finding confirmed an enhanced affinity of the amino acceptor in the mutant M9-1. As a result, the distances between the N atom on amino group of PMP and the O atom on ketone group of d-fructose were gradually reduced to less than 3 Å (Figure 6D), which facilitated the occurrence of transamination reaction. The hydrogen bonds were frequently formed between the sidechain of R69 and d-fructose (Figure 6E), suggesting the advantage of the replacement of lysine by arginine.

**Table 3:** Kinetic parameters of purified WT, M9, and M9-1 toward (R)-MBA and d-fructose

Substrate	Enzyme	*K*_*m*_ (mM)	*k*_*cat*_ (min^−1^)	*k*_*cat*_*/K*_*m*_ (min^−1^mM^−1^)
(R)-MBA	WT	0.71	0.76	1.07
	M9	3.93	11.32	2.88
	M9-1	8.20	38.99	4.75
d-fructose	WT	–	–	–
	M9	37.11	48.97	1.32
	M9-1	22.19	56.52	2.55

Conversely, the affinity for the amino donor in both M9 and M9-1 was weakened as a result of the protein engineering (Table 3), consistent with the observed reduction in the number of hydrogen bonds (Figure S8). However, the amino donor in the mutant showed a closer proximity to PLP due to a π-cation reaction between R145 and benzene ring of (R)-MBA. Significantly, the introduction of H69R and K145R mutations in M9-1 achieved a remarkable balance in the binding of the amino donor and acceptor, which provides insights into the protein engineering of enzymes with ping-pong mechanism.

### *In vitro* enzyme cascade reaction for the preparation of azasugar

The maximum conversion rates of MvTA WT and M9-1 to four hexaketoses (d-fructose, d-allulose, d-tagatose, d-sorbose) were determined. The results demonstrated a significant enhancement in the maximum conversion rates of M9-1 compared to WT (Table 4). Notably, M9-1 exhibited a maximum conversion rate of over 50% for d-fructose. Mass spectrometric analysis confirmed the transamination products of the four hexaketoses, with molecular weights of 182.1 Da [M + H]^+^, providing evidence of the mutant M9-1’s capability to catalyze the transamination reaction of these hexaketoses (Figure S6).

**Table 4:** Maximum conversion rate of WT and mutant M9-1 toward d-hexaketoses

Substrate	Product	Conversion rate of WT (%)	Conversion rate of M9-1 (%)
d-fructose	2-amino-2-deoxy-d-mannitol, **7**	28.09	50.64
d-tagatose	5-amino-5-deoxy-d-altritol, **9**	18.83	30.72
d-allulose	2-amino-2-deoxy-d-altritol, **8**	9.39	29.99
d-sorbose	2-amino-2-deoxy-d-iditol, **10**	5.41	11.12

**Figure 7:**
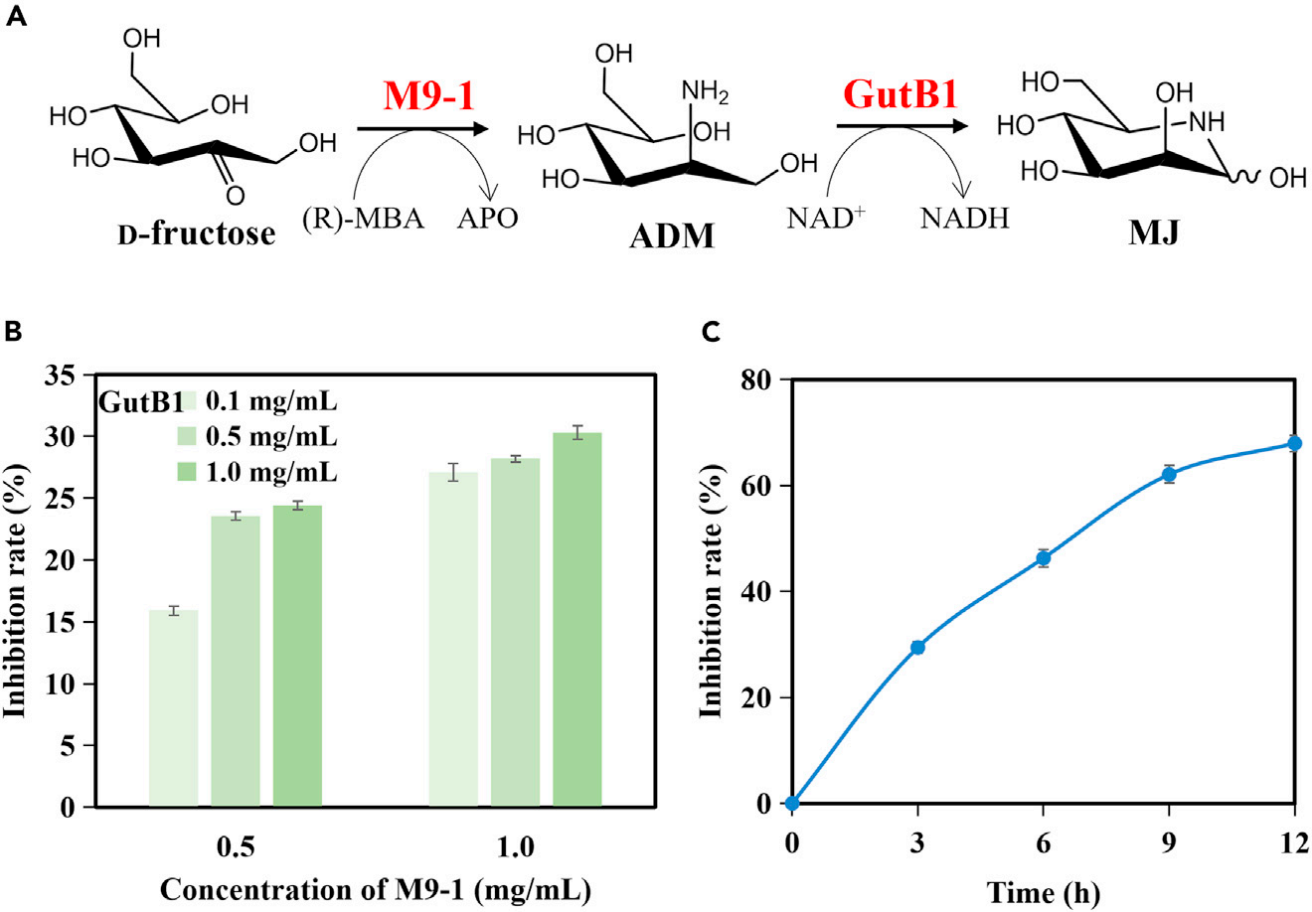
Establishment of an enzyme cascade reaction for biosynthesis of azasugars (A) Biosynthesis pathway for azasugars, such as MJ, 1-DMJ and 1-DNJ. (B) Optimization of enzyme dosage of transaminase M9 and dehydrogenase GutB1 for MJ preparation using d-fructose as the substrate. (C) Process of the enzyme cascade reaction.

An *in vitro* enzyme cascade method was developed for the preparation of azasugar, employing M9-1 and the dehydrogenase GutB1 derived from *Paenibacillus polymyxa* (Figure 7A). When d-fructose was utilized as the substrate, the product MJ was quantified through the inhibition rate of glucosidase. Optimization of enzyme amounts and the M9-1-to-GutB1 ratio revealed that the transaminase played a crucial role as the rate-limiting enzyme, requiring a high concentration (1.0 mg/mL). Increasing the GutB1 concentration did not yield significant improvements in MJ yield, thus establishing an M9-1-to-GutB1 ratio of 10:1 (Figure 7B). As the reaction time extended, the inhibition rate of the reaction product gradually increased, indicating the accumulation of MJ. After 9 h of reaction, the increase in product inhibition rate showed a diminished trend. (Figure 7C). Subsequently, MJ could be further converted into 1-DNJ through epimerization, dehydration, and reduction reactions. In the microbial biosynthetic pathway of 1-DNJ,^13^ the transaminase GabT1 catalyzed the transamination reaction of fructose 6-phosphate (6-P-Fru), requiring subsequent dephosphorylation by YktC1 before dehydrogenation to generate ADM. By utilizing M9-1 for d-fructose transamination, the dephosphorylation step could be circumvented, and the cost of d-fructose is significantly lower compared to 6-P-Fru, rendering the *in vitro* enzyme cascade reaction more economical and efficient.

### Conclusion

The MvTA was a suitable enzyme for the *in vitro* production of 1-DNJ through the initial transamination reaction of d-fructose. However, due to its low activity and thermostability, protein engineering was employed to enhance its performance. The obtained mutant M9, incorporating nine mutation sites, exhibited a remarkable 16.4-fold increase in specific activity and an impressive 208-fold improvement in thermostability compared to WT. Through further rational design using the crystal structure of M9, the mutant M9-1 with an additional 1.9-fold improvement in specific activity was obtained. MD simulations analysis indicated that the mutation sites H69R and K145R in the flexible loop of M9-1 were found to play crucial roles in the binding of the amino acceptor and donor, respectively. Together with other mutant sites such as L162I and K142P, the substrates achieved a more stable conformation within the active pocket and better access channels, facilitating the two-stage ping-pong reaction of transamination. The M9-1 mutant was combined with the dehydrogenase GutB1 to develop an enzyme cascade reaction for the *in vitro* production of mannojirimycin (MJ), a precursor of 1-DNJ. Furthermore, given the improved catalytic activity of M9-1 toward various hexaketoses, the synthesis of a range of azasugars might be explored, enabling the preparation of end products with desirable biological functions.

### Limitations of the study

In this study, we successfully obtained the precursor of 1-DNJ (MJ) through an *in vitro* enzyme cascade reaction. However, several enzymes crucial to 1-DNJ biosynthesis remain unidentified. Consequently, our upcoming efforts will concentrate on discovering these unknown enzymes to establish an external synthesis pathway for 1-DNJ. Additionally, we aim to devise a quantitative detection method to accurately measure MJ yield, supplanting the glycosidase inhibition rate approach in this study.

## STAR★Methods

### Key resources table

**Table:** 

REAGENT or RESOURCE	SOURCE	IDENTIFIER
**Bacterial and virus strains**		

*Escherichia coli* DH5α	Tsingke	TSC-C01
*Escherichia coli* BL21	Tsingke	TSC-E01

**Recombinant DNA**		

pET21a-MvTA	This paper	N/A
pET21a-GutB1	This paper	N/A

**Software and algorithms**		

Autodock Vina	N/A	https://vina.scripps.edu/
Gromacs 2020	N/A	https://www.gromacs.org/
AmberTools22	N/A	https://ambermd.org/AmberTools.php
Rosetta	N/A	https://www.rosettacommons.org/software

**Chemicals, peptides, and recombinant proteins**		

MvTA (No. WP_011781668)	NCBI	https://www.ncbi.nlm.nih.gov
GutB1 (No. CP002213, 2685402–2686451)	NCBI	https://www.ncbi.nlm.nih.gov

### Resource availability

#### Lead contact

Further information and requests for resources and reagents should be directed to and will be fulfilled by the lead contact, Professor Yuanxia Sun (sun_yx@tib.cas.cn).

#### Materials availability

This study did not generate new reagents.

#### Data and code availability

All data reported in this paper are available within the paper and the supplemental information files. The MvTA and GutB1 protein sequences used in this work were downloaded from NCBI database (www.ncbi.nlm.nih.gov). Three crystal structures of MvTA (PDB ID: 8IOZ, 8ISC and 8IVP) have been published in PDB database (www.rcsb.org). Data will be shared by the lead contact upon request.

This paper does not report original code.

Any additional information required to reanalyze the data reported in this paper is available from the lead contact upon request.

### Experimental model and subject details

*Escherichia coli* DH5α strain was used to prepare the plasmids and construct mutants. The cells were incubated in Luria−Bertani (LB) medium containing 100 μg/mL ampicillin at 37°C. *E. coli* BL21(DE3) strain was used to express the target protein. The cells were grown in LB medium containing 100 μg/mL ampicillin at 37°C until the OD_600_ reached 0.6–0.8. Protein expression was induced by adding 0.5 mM IPTG at 25°C for 20 h.

### Method details

#### Materials

The d-fructose, d-allulose, d-sorbose and d-tagatose were purchased from Sigma-Aldrich (St Louis, MO). All other reagents were of analytical grade and obtained from Aladdin (Shanghai, China). The wild-type genes of MvTA and GutB1 were synthesized by GENEWIZ (Suzhou, China).

#### High-throughput screening method

The single clones from mutant libraries were cultivated in 96-deep-well plates with Luria−Bertani medium supplemented with 100 μg/mL ampicillin at 37°C. Protein expression was induced by adding 0.5 mM IPTG to the medium. After 24 h of incubation, cells were centrifuged at 5000 rpm for 20 min and resuspended in phosphate buffered saline (PBS; 100 mM, pH 7.0) containing 1 mg/mL lysozyme. The suspension was incubated at 37°C for 1 h, followed by cell disruption using a freeze-thaw method. Supernatants were obtained by centrifugation at 5000 rpm for 30 min and transferred to 96-well plates for the transamination reaction. The reaction system included 25 mM 4-nitrophenethylamine, 10 mM d-fructose, and 0.1 mM pyridoxal phosphate (PLP). After incubating at 30°C for 12 h, the absorbance at 500 nm (OD_500_) was measured using a BioTek Synergy LX Multi-Mode Reader (Agilent, US). Clones with higher OD_500_ values than the wild type were selected for further analysis.

#### Site-directed saturation mutagenesis and random mutagenesis

Site-directed saturation mutagenesis was performed using PCR with primers listed in Table S1, and the NDT/VHG/TGG codon strategy was employed to generate less-redundant libraries.^31^ The plasmid pET21a-MvTA containing the wild-type MvTA gene was used as the PCR template. The PCR products were digested by the *Dpn*I enzyme and transformed into *E. coli* BL21 (DE3) to generate the mutation libraries. Random mutagenesis was carried out using GeneMorph Ⅱ Random Mutagenesis Kit (Agilent, US).

#### Enzyme expression and purification

Candidate clones selected through high-throughput screening were cultivated in LB medium supplemented with 100 μg/mL ampicillin at 37°C until the OD_600_ reached approximately 0.6–0.8. Protein expression was induced by adding 0.5 mM IPTG at 25°C for 20 h. Cells were harvested by centrifugation at 6000 rpm for 10 min, suspended in PBS, and disrupted by sonication. The supernatant was obtained by centrifugation at 14,000 rpm at 4°C for 20 min and used for protein purification. Recombinant proteins were purified using a BeaverBeads IDA-Nickel Kit (Beaver, Suzhou, China). Magnetic beads were equilibrated with the binding buffer (100 mM PBS, 500 mM NaCl, 20 mM imidazole, pH 7.0), added to the supernatant, and incubated at 4°C for 30 min to bind the recombinant protein. The target proteins were eluted using elution buffer (100 mM PBS, 500 mM NaCl, 500 mM imidazole, pH 7.0), and then concentrated and desalted using Amicon Ultra-15 centrifugal filters (Millipore, USA). Protein concentration was determined using the Bradford protein kit with bovine serum albumin as the standard.

#### Enzymatic activity and thermostability assays

The activities of MvTA and its mutants were assayed in 100 mM PBS (pH 6.0) containing 0.1 mg/mL purified enzyme, 0.1 mM PLP, 25 mM (R)-α-methylbenzylamine ((R)-MBA) as amino donor, and 10 mM d-fructose as amino acceptor. Reactions were performed at 50°C for 2 h and terminated by adding an equal volume of acetonitrile:HCl (19:1) solution. One unit (U) of enzymatic activity was defined as the amount of enzyme catalyzing the production of 1 μmol acetophenone (APO) from (R)-MBA. The APO product was analyzed using an HPLC system equipped with a C18 column (4.6 mm × 250 mm, Welch, Shanghai, China) and an ultraviolet detector at 254 nm. The mobile used was solution A (acetonitrile with 0.1% formic acid) and solution B (H_2_O with 0.1% formic acid), with a flow rate of 1 mL/min and the following gradient: 0–15 min, 30–80% A.

For the determination of thermostability, WT and mutant enzymes were incubated in 100 mM PBS (pH 6.0) at temperatures of 30, 40, 50°C and 60°C, respectively. Residual activities were measured at different time intervals using HPLC. The half-life (t_1/2_) was calculated based on the first-order deactivation function: ln(E/E0) = −k_d_t; t_1/2_ = ln 2/k_d_, where k_d_ represents the deactivation rate constant.

#### Kinetic parameter measurement

The kinetic parameters of WT and the best mutant toward (R)-MBA and d-fructose were determined, respectively. Reaction mixtures containing PBS buffer (100 mM, pH 6.0), 0.1 mM PLP, 20 mM d-fructose, wild-type (0.2 mg/mL) or mutant enzyme (0.1 mg/mL), and varying concentrations (1, 2, 5, 15, 20, and 25 mM) of (R)-MBA were incubated at 30°C for 2 h. The *K*_*m*_ and *k*_cat_ values were calculated by nonlinear regression fitting (saturation hyperbola model) using GraphPad Prism 7.0. All kinetic parameters were measured in triplicate.

#### Crystallization, data collection, structure determination and refinement

All crystallization experiments were conducted at 25°C using the sitting-drop vapor-diffusion method. Typically, 1 μL of WT or M9 (10 mg/mL) was mixed with 1 μL of reservoir solution in 48-well Cryschem Plates and equilibrated against 100 μL of the reservoir at 25°C. Crystals of WT-apo were obtained using condition No. 47 (32% v/v Tacsimate pH 7.0, 0.1 M BIS-TRIS propane pH 7.0) from the SaltRx II screen kit (Hampton Research). Dehydration was performed to improve the diffraction resolution of the WT-apo crystal, using 20% glycerol for dehydration for less than 1 h. Crystals of M9-apo were obtained using condition No. 2.4 (1.26 M ammonium sulfate, 0.2 M lithium sulfate 0.1 M Tris, pH 8.5) from the JCSG screen kit (MD). For the complex structures of M9 with ligands, the crystals were soaked in a liquid containing PLP and d-fructose or PLP and (R)-MBA for less than 1 h.

X-ray diffraction data were collected and tested at beamlines BL10U2, BL17B, BL18U1 and BL19U1 of National Facility for Protein Science in Shanghai (NFPS) at Shanghai Synchrotron Radiation Facility (SSRF). The crystals were mounted in a Cryoloop and soaked with a cryoprotectant solution prior to data collection at 100 K. Diffraction images were processed using HKL2000.^32^ Crystal structures were solved by molecular replacement (MR) using the Phaser program^33^ from the Phenix^34^ suite, with the structure of PHYRE2 used as the search model. Further refinement was performed using the phenix.refine package^35^ and Coot.^36^ Prior to structural refinements, 5% randomly selected reflections were set aside for calculating *R*_*free*_^37^ as a monitor.

#### Molecular docking and molecular dynamics simulation

(R)-MBA was docked into the M9-PLP crystal structure using the AutoDock Vina program,^38^^,^^39^ resulting in complex structure of M9-PLP-MBA. M9 was mutated back to WT to predict the wild-type structure using the PyMOL mutagenesis tool. PLP and (R)-MBA were sequentially docked into the WT structure to obtain the complex structure WT-PLP-MBA. The complex structures of WT-PMP-APO, M9-PMP-APO, M9-PMP-FRU, and M9-1-PMP-FRU, were obtained using the same method.

To explain the possible mechanism for improved catalytic activity, molecular dynamics (MD) simulations were performed on the complex structures using GROMACS 2020 program. The ff14SB force field was used to model the protein, and small molecule topologies were generated by ACPYPE.^40^ The complex structures were solvated in TIP3P^41^ with a cubic water box and neutralized by adding Na^+^ counterions. Energy minimization and equilibration were performed, followed by an unconstrained MD simulation of 100 ns at 298.15 K and 1 atm with a 2 fs integration time step. PyMOL was used for visualization and to construct graphical illustrative figures.

#### Further enhancement of activity through rosetta cartesian-ddg

To achieve further enhancement of activity, rational design was performed using the Rosetta Cartensian_ddg application.^42^ The M9 crystal structure was utilized as the starting point, and both PMP and d-fructose were docked into the structure to form the complex M9-PMP-Fru, serving as the initial atomic coordinate. The complex structure underwent relaxation in both internal and Cartesian coordinate. Subsequently, the relaxed structure was subjected to Cartensian_ddg calculation. The residues within 6 Å radius of d-fructose were computationally designed to point mutations with ΔΔG values below −1.0. These designed mutations were then validated through activity assays. The gmx_MMPBSA tool was employed to calculate the binding free energy between the protein and d-fructose.^43^

#### Transamination reaction with hexaketoses as substrate

To determine the maximum conversion rate of WT and M9-1 toward hexaketoses (d-fructose, d-allulose, d-sorbose and d-tagatose), reaction mixtures containing PBS buffer (100 mM, pH 6.0), 0.1 mM PLP, 10 mM hexaketose, 25 mM (R)-MBA, and wild-type or mutant enzyme (1.0 mg/mL) were incubated at 30°C for 48 h. The conversion rates of different hexaketoses were estimated based on the conversion rates of APO. The concentrations of produced APO were derived from the standard APO curve, and the conversion rates were computed by dividing the APO concentration by the total (R)-MBA concentration. Transamination products of hexaketoses were identified using electrospray ionization mass spectrometry (ESI-MS).

#### Establishment of *in vitro* enzyme cascade for manufacturing azasugar

The *in vitro* enzyme cascade method was established using M9-1 and the *Paenibacillus polymyxa* dehydrogenase GutB1, which was expressed by *E. coli* BL21 (DE3). The reaction system contained PBS buffer (100 mM, pH 6.0), 0.1 mM PLP, 10 mM NAD^+^, 10 mM d-fructose, 25 mM (R)-MBA, and both enzymes (M9-1 and GutB1). The concentrations of the M9-1 and GutB1 were optimized by adding different amounts of enzymes. The mannojirimycin (MJ) product was quantified using a glucosidase inhibition assay, as previously described by Jiang et al.^44^

### Quantification and statistical analysis

Protein concentration was determined using the Bradford protein kit on BioTek synergy LX multi-mode reader. For enzyme assay of MvTA, the yield of acetophenone (APO) was determined and analyzed using Agilent 1260 Infinity II HPLC system. The mannojirimycin (MJ) product was quantified using the glucosidase inhibition assay on BioTek synergy LX multi-mode reader.

## Supplemental information

Document S1. Figures S1–S9 and Table S2

Table S1. List of primers used in this study, related to STAR Methods
