# Supplementary material for: Protein engineering of transaminase facilitating enzyme cascade reaction for the biosynthesis of azasugars
Source: iScience. 2024 Jan 26;27(3):109034. doi: 10.1016/j.isci.2024.109034 (PMC10904899; doi:10.1016/j.isci.2024.109034)
Supplement: Document S1. Figures S1–S9 and Table S2 [file mmc1.pdf]

iScience, Volume *27*

Supplemental information

Protein engineering of transaminase facilitating enzyme cascade reaction for the biosynthesis of azasugars

Yueming Zhu, Peng Chen, Qianzhen Dong, Qian Li, Dechuan Liu, Tao Liu, Weidong Liu, and Yuanxia Sun

Supplemental Information

Content

Table S2. Data collection and refinement statistics of WT and M9 mutant crystals

Figure S1. Enzymatic properties of wild-type MvTA

Figure S2. Relative thermostability of single-site mutants

Figure S3. Comparison of enzymatic properties between WT and M9

Figure S4. Overall structure of the WT protein

Figure S5 The B-factor comparation between the structure of 8ISC and 8IVP

Figure S6. MD simulation analysis of WT and M9

Figure S7. RMSF profiles of the protein residues during the MD simulation

Figure S8. Number of hydrogen bonds between the protein and substrate during the MD simulation

Figure S9. Identification of molecular weight of transamination products using four D-hexaketoses as amino acceptor by ESI-MS

Table S2. Data collection and refinement statistics of WT and M9 mutant crystals, related to Figure 4. Values in parentheses are for the outermost resolution shells.

	**WT**	M9-PLP	M9-PLP-Fru
Data collection			
**Space group**	*P6₅22*	*P2₁*	*P2₁*
**Unit-cell**			
a, b, c **[Å]**	71.443, 71.443, 499.721	80.950 ,112.850, 92.244	80.707 ,112.243, 92.098
α /β /γ **(°)**	90/90/120	90/112.372/90	90/112.313/90
**Resolution (Å)**	50-2.33 (2.41-2.33)	50-2.27 (2.41-2.27)	50-1.93 (1.99-1.93)
**Unique reflections**	34208 (3307)	66664 (6865)	106452 (10275)
**Redundancy**	40.4 (38.7)	5.4 (5.0)	6.3(6.2)
**Completeness (%)**	99.9 (100)	95.0 (97.9)	93.2 (90.6)
**Average I/σ(I)**	15.9(1.7)	15.0 (3.2)	18.1(3.4)
**CC 1/2**	0.996（0.799）	0.991 （0.909）	0.991(0.767)
Refinement			
**R_{work} (95% data)**	22.0 (26.6)	19.3 (26.6)	15.1(21.6)
**R_{free} (5% data)**	26.0 (31.4)	24.7 (36.3)	18.8(26.5)
**Rmsd bonds (Å)**	0.002	0.015	0.016
**Rmsd angles (°)**	0.561	1.970	1.930
**Dihedral angles**			
**Most favored (%)**	94.2	96.1	97.5
**Allowed (%)**	4.2	3.89	2.5
**Disallowed (%)**	1.6	0.0	0.0
**Protein**	4500/55.15	9925/20.38	9224/16.56
**Water**	99/47.8	1156/24.70	1538/27.49
**Ligand**	-	96/20.46	144/22.25
PDB ID code	8IOZ	8ISC	8IVP

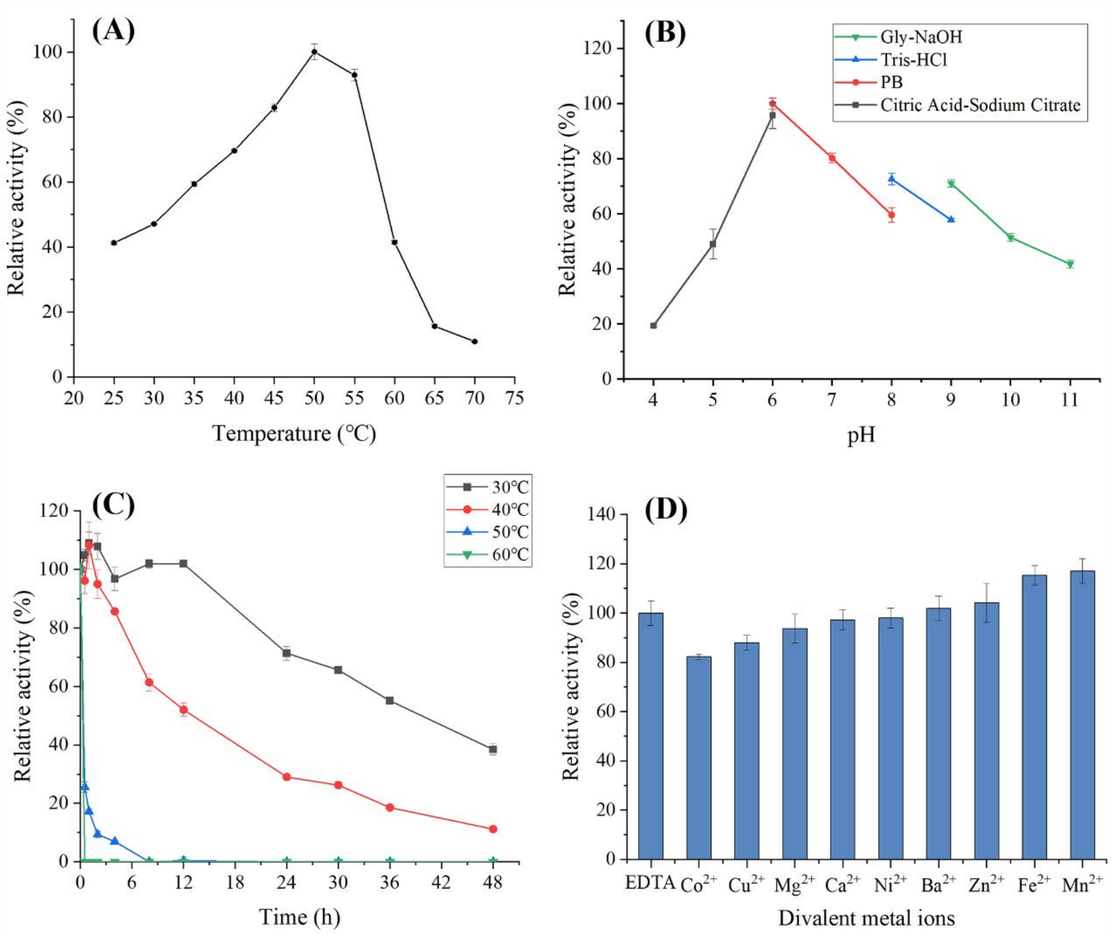

Figure S1. Enzymatic properties of wild-type MvTA, related to Figure 2. (A) Effect of temperature on enzyme activity. (B) Effect of pH on enzyme activity. (C) Residual activities of MvTA after incubated at different temperatures. (D) Effect of metal ions on enzyme activity.

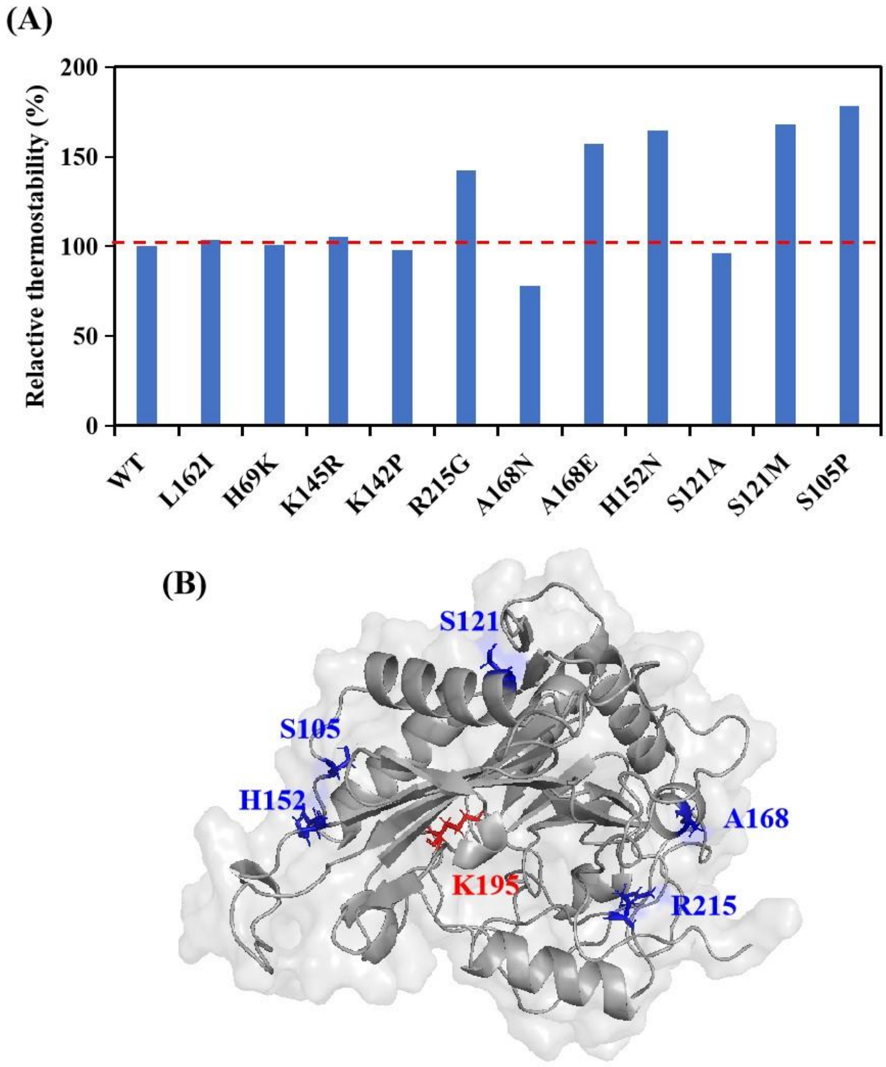

Figure S2. Mutation sites involved in thermostability, related to Figure 2. (A) Relative thermostability of single-site mutants. Purified mutant enzymes were treated at 55 °C for 2 h, and residual activities were measured. Relative thermostability was calculated by setting the WT enzyme activity after incubation as 100%. (B) Location of amino acid residues influencing the thermostability of MvTA.

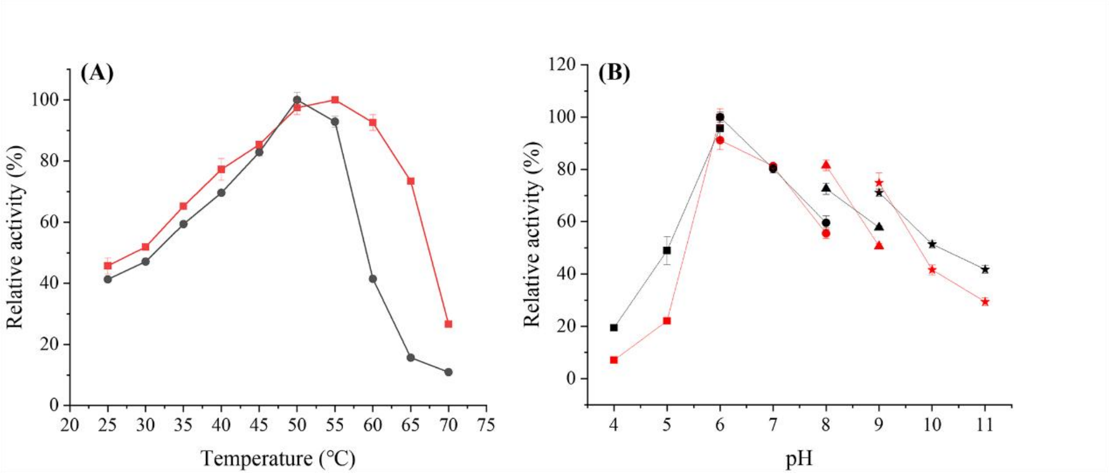

Figure S3. Comparison of enzymatic properties between WT (black) and M9 (red), related to Figure 3. (A) Effect of temperature on enzyme activities. (B) Effect of pH on enzyme activities.

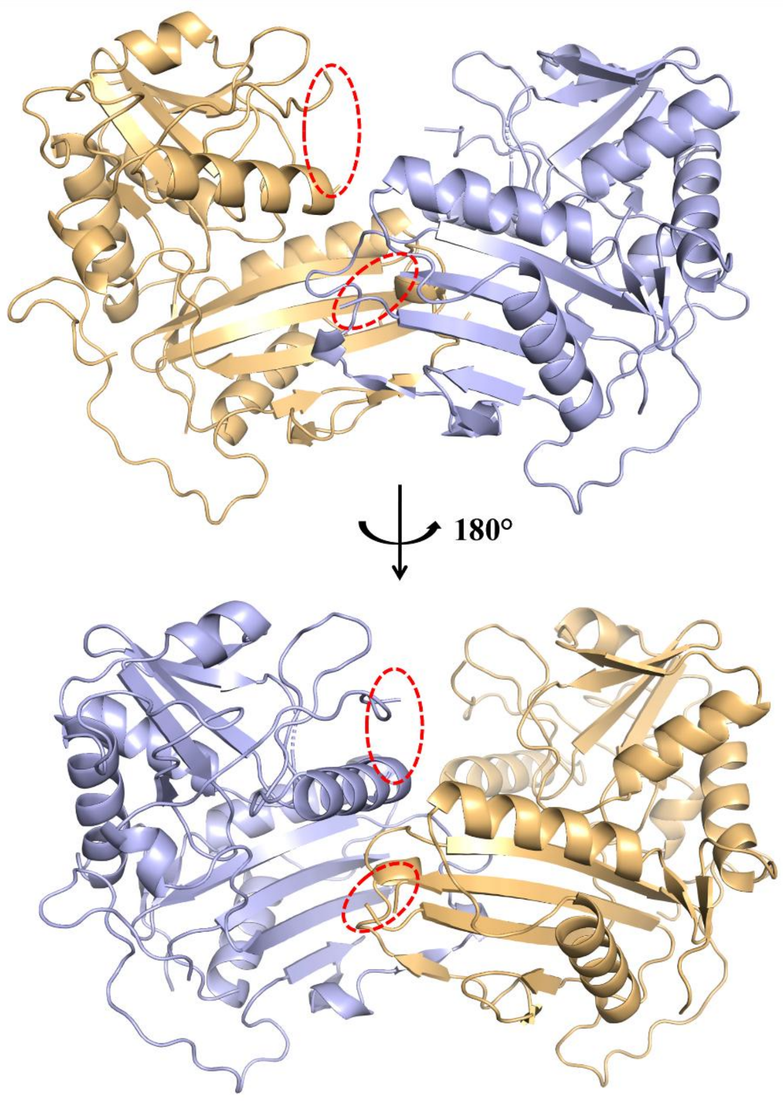

Figure S4. Overall structure of the WT protein, related to Figure 4. The four missing loops are highlighted by red circles.

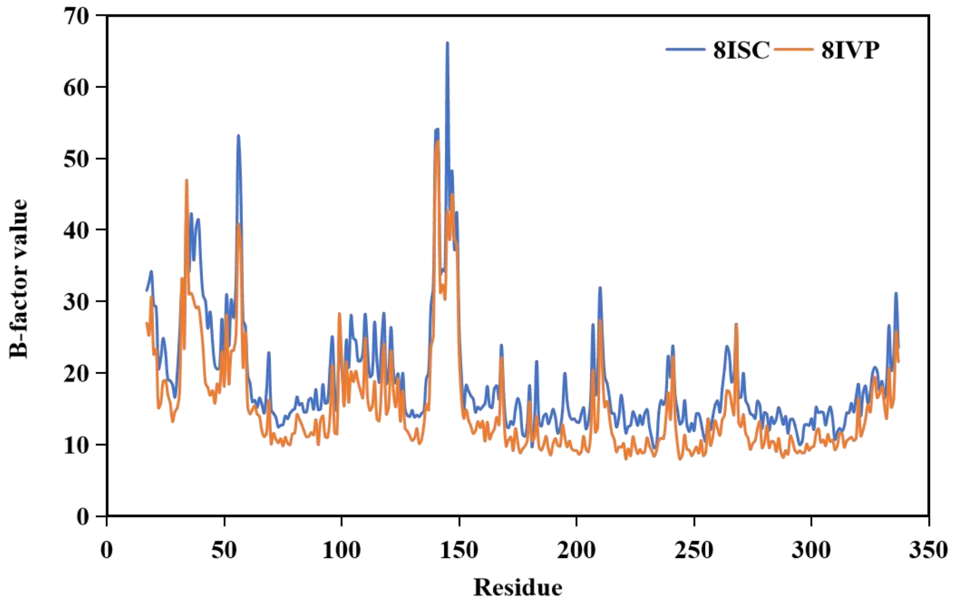

Figure S5. The B-factor comparation between the structure of 8ISC and 8IVP, related to Figure 4.

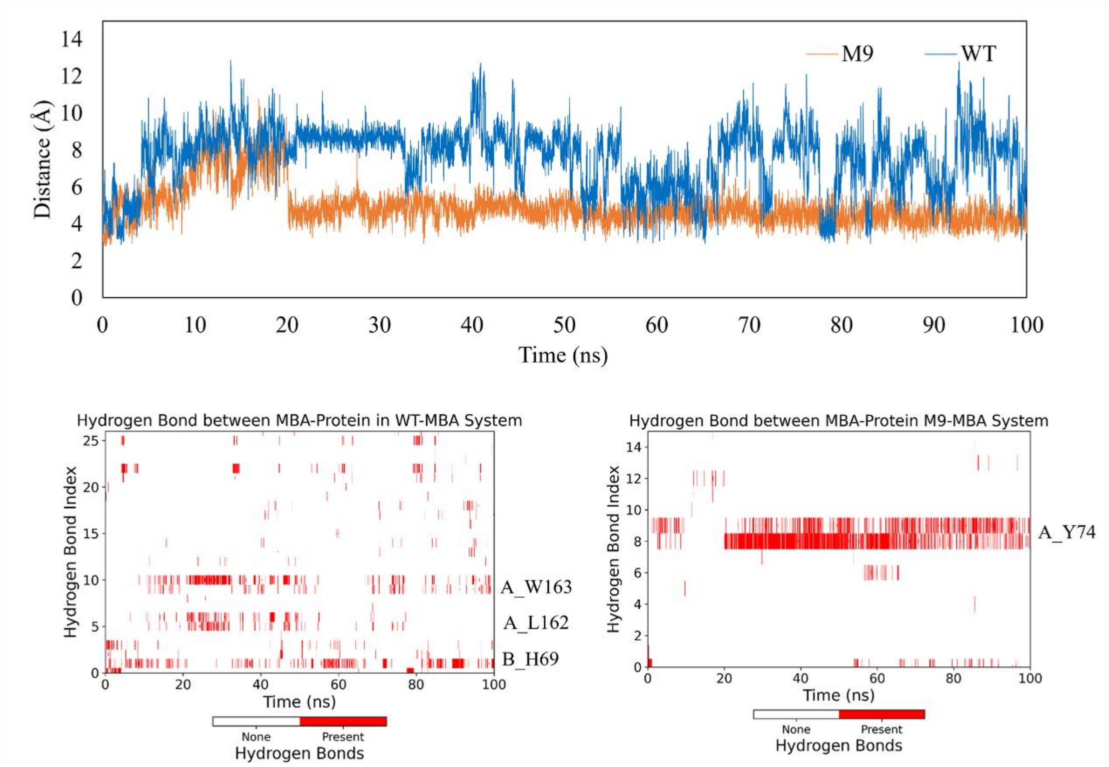

Figure S6. MD simulation analysis of WT and M9, related to Figure 5. (A) Distance between the N atom of (R)-MBA and the O atom of PLP during 100 ns trajectory. (B) Hydrogen bonds between (R)-MBA and protein in the WT-PLP-MBA complex. (C) Hydrogen bonds between (R)-MBA and protein in the M9-PLP-MBA complex.

(A)

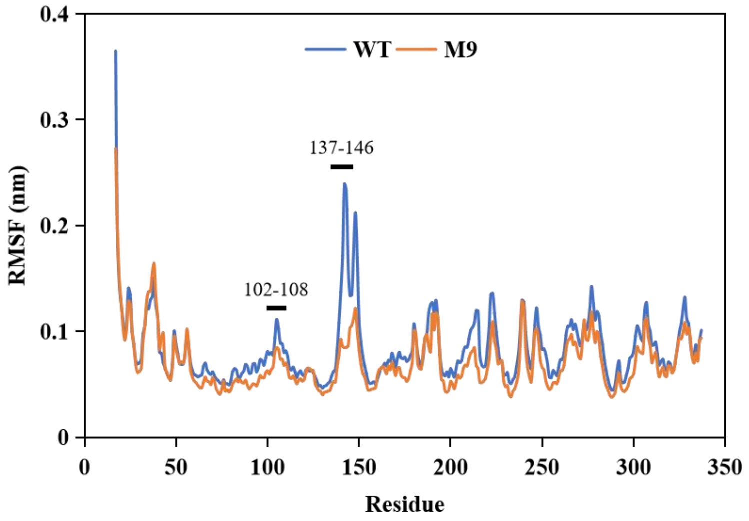

(B)

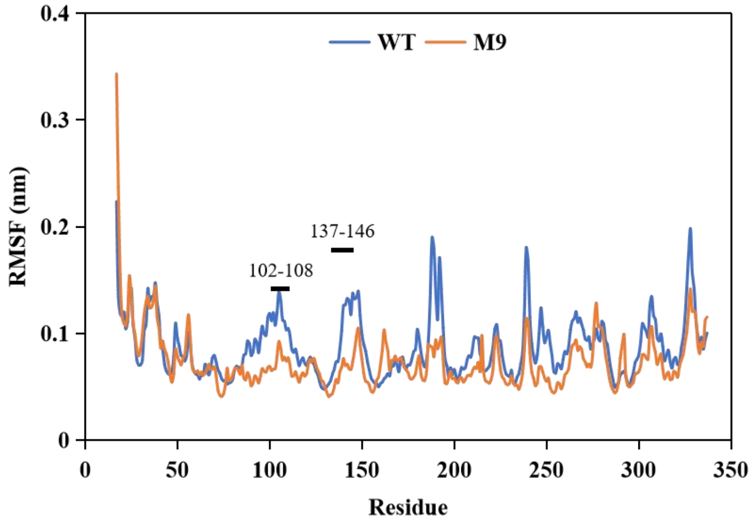

Figure S7. RMSF profiles of the protein residues during the MD simulation, related to Figure 5. (A) RMSF profile of the protein residues during the MD simulation of WT-PLP-MBA and M9-PLP-MBA complex. (B) RMSF profile of the protein residues during the MD simulation of WT-PMP-Fru and M9-PMP-Fru complex.

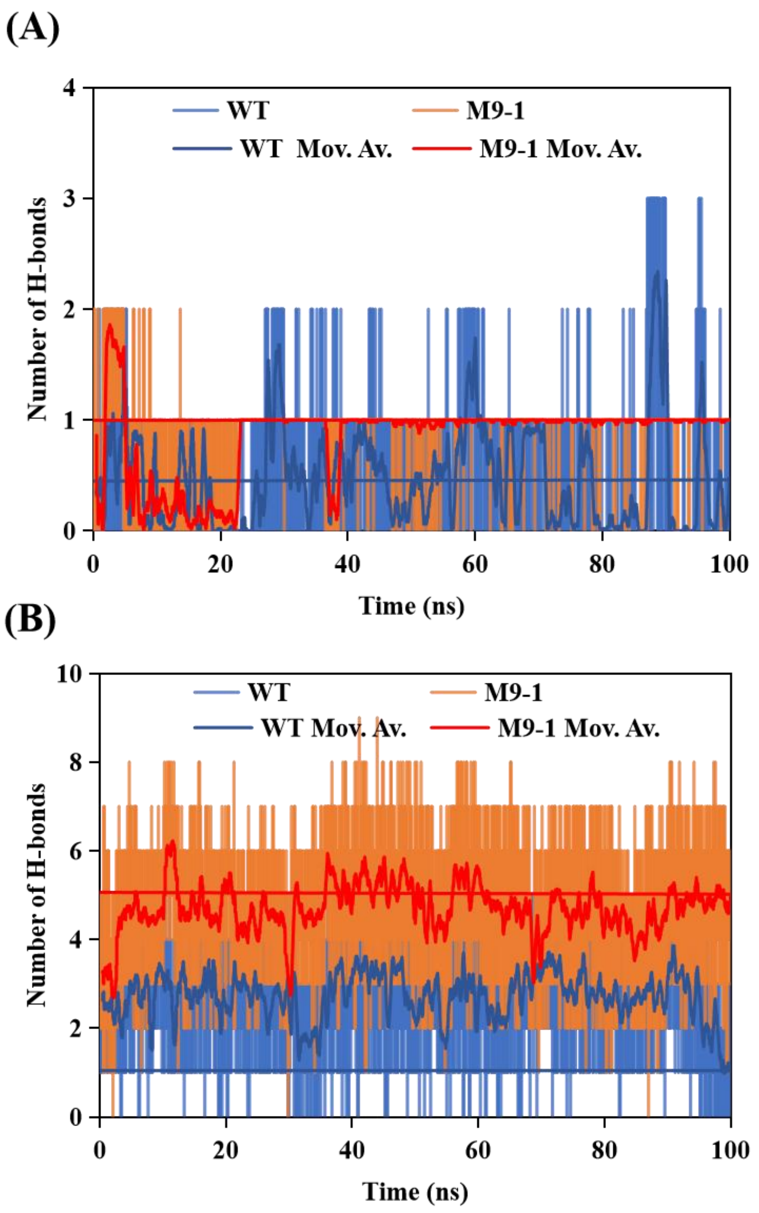

Figure S8. Number of hydrogen bonds between the protein and substrate during the MD simulation, related to Figure 6. (A) Number of hydrogen bonds between the protein and (R)-MBA during the MD simulation. (B) Number of hydrogen bonds between the protein and fructose during the MD simulation.

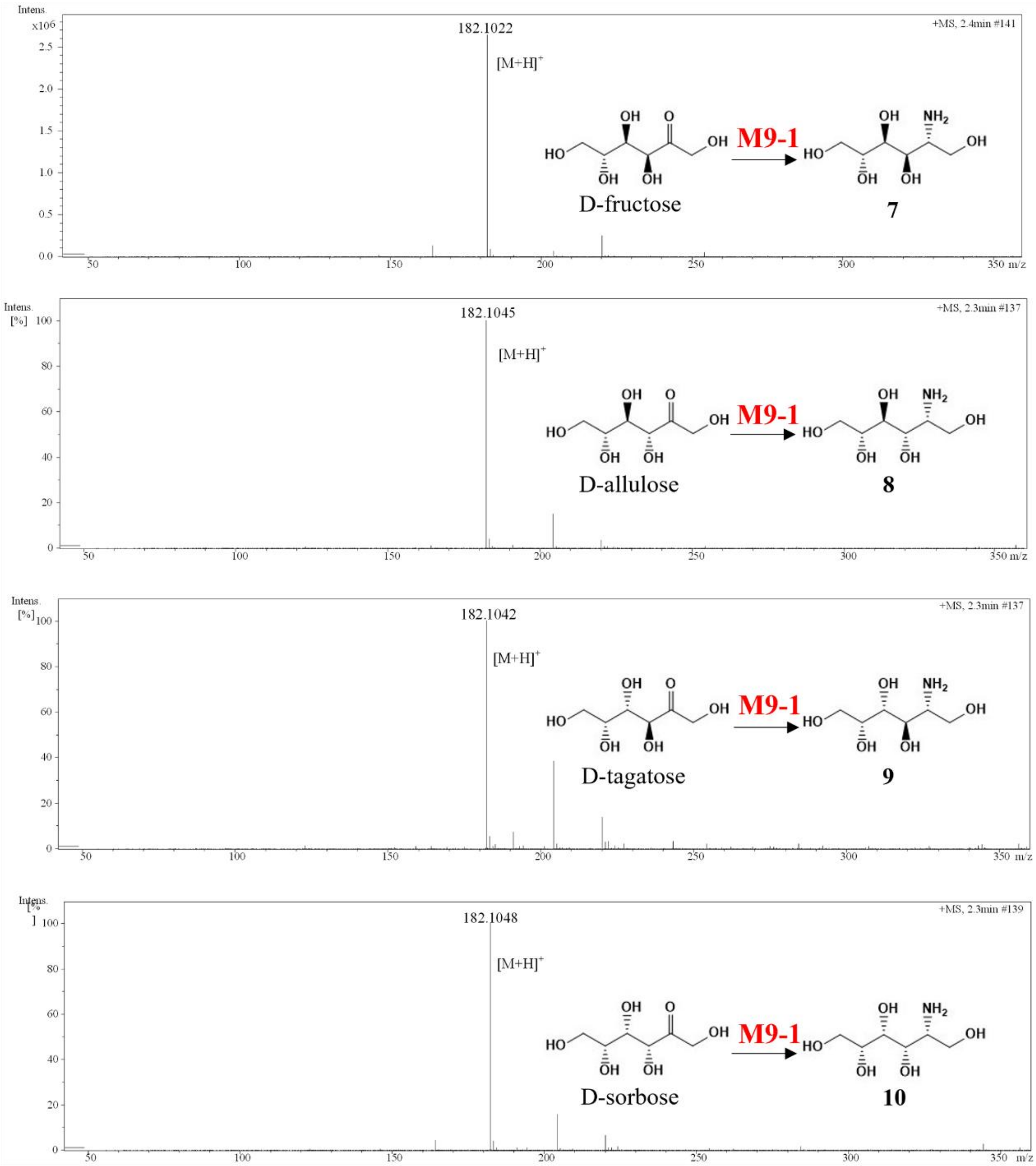

Figure S9. Identification of molecular weight of transamination products using four D-hexaketose as amino acceptors by ESI-MS, related to Table 4.
